# Maternal Deprivation Enhances Behavioral Vulnerability to Stress Associated with miR-504 Expression in Nucleus Accumbens of Rats

**DOI:** 10.1371/journal.pone.0069934

**Published:** 2013-07-26

**Authors:** Yi Zhang, Xiongzhao Zhu, Mei Bai, Li Zhang, Liang Xue, Jinyao Yi

**Affiliations:** 1 Medical Psychological Institute, Second Xiangya Hospital, Central South University, Changsha, Hunan, China; 2 Key Laboratory of Psychiatry and Mental Health of Hunan Province, Central South University, Changsha, Hunan, China; 3 Hunan Province Technology Institute of Psychiatry, Central South University, Changsha, Hunan, China; Radboud University, The Netherlands

## Abstract

**Objective:**

In this study, the effect of maternal deprivation (MD) and chronic unpredictable stress (CUS) in inducing depressive behaviors and associated molecular mechanism were investigated in rats.

**Methods:**

Maternal deprivation was established by separating pups from their mothers for 6 hours daily from postnatal day 1 to day 14. Chronic unpredictable stress was established by water deprivation, elevated open platform, food deprivation, restraint stress and electric foot shock. The depressive behaviors were determined by use of sucrose preference test and forced swim test.

**Results:**

Rats in MD/CUS group exhibited lower sucrose preference rate, longer immobility time, and lighter body weights than rats in other groups (MD/control, non-MD/CUS and non-MD/control group). Meanwhile, higher miR-504 expression and lower dopamine receptor D1 (DRD1) and D2 (DRD2) expression were observed in the nucleus accumbens of rats in the MD/CUS group than in the other three groups. MiR-504 expression correlated negatively with DRD1 gene expression and sucrose preference rate in the sucrose preference test, but correlated positively with immobility time in forced swim test. Both DRD2 mRNA and protein expression correlated negatively with immobility time in forced swim test.

**Conclusion:**

These results suggest that MD enhances behavioral vulnerability to stress during adulthood, which is associated with the upregulation of miR-504 and downregulation of DRD2 expression in the nucleus accumbens.

## Introduction

Early-life stress is one of the most important adverse events associated with an increased risk of developing depression and anxiety disorders [1], [2]. Individuals exposed to adverse events during early life are more prone to developing depression-like behavior upon exposure to stress in adulthood [3], [4]. However, little is known about the molecular mechanism underlying behavioral vulnerability to chronic stress induced by early-life stress. A large number of preclinical studies in animal models and clinical studies have consistently revealed that adverse experiences in early life can affect long-term behavior and function of the neurotransmission system during adult life [5], [6]. One of the established animal models for analyzing the influence of early life adversity is maternal deprivation in rats, which is important for regulating the development of the neurotransmission system as well as brain functions and emotional behaviors during adulthood [7], [8]. Therefore, we hypothesize that the dysfunction of the neurotransmission system induced by maternal deprivation may increase behavioral vulnerability to stress in adults.

Mesolimbic dopamine system is one of the key neurotransmission systems in the brain, which play an important role in emotional responses to stress [3], [9]. Several lines of evidence have shown that early life stress affects the development of dopaminergic neurons and the expression of dopamine receptor genes leading to consequent dysfunction of the dopamine system during adulthood [3]. Dopamine receptor D1 (DRD1) and D2 (DRD2) are key dopamine receptors that regulate growth and differentiation of dopaminergic neurons and are abundantly distributed in the nucleus accumbens (NAc) [10], [11]. Previous studies showed that DRD1 expression decreased during exposure to stressors in rat models. For example, stressors-induced anhedonia-like behaviors in rats have been demonstrated to be associated with decreased dopaminergic response to tasty food and decreased sensitivity of DRD1 in the nucleus accumbens (NAc) [12]. Furthermore, electroconvulsive therapy (ECT) in depressive rhesus monkey models resulted in transient increases in DRD1 expression [13]. In rodent models, selective DRD1 agonists significantly inhibited antidepressant-like effects in both learning helplessness procedure and forced swim test [14], [15]. Our previous study demonstrated that DRD2 expression was significantly downregulated in the striatum of MD and CUS rats, which significantly correlated with depression-like behaviors [3]. However, the molecular mechanisms underlying decreased DRD1 and DRD2 expression in stress-induced depression have yet to be fully elucidated.

MicroRNAs (miRNAs) are small (∼22 bp) single-stranded, noncoding RNAs involved in important post-transcriptional mechanisms [16], [17]. Aberrant miRNAs expression has been observed in stress-induced depressive rats, which includes maternally deprived (MD) rats [4], [18]. A recently in vitro study showed that miR-504 up-regulates DRD1 expression by binding directly to the 3′-UTR of the *DRD1* gene [19]. In this study, we investigated miR-504 and dopamine receptor D1/D2 expression in the NAc of rats that experienced MD and chronic unpredictable stress (CUS) as well as behavioral consequences of early life MD on CUS exposure during adulthood.

## Materials and Methods

### 1. Animals

Adult pregnant Sprague–Dawley rats (SLACLABORATORY ANIMAL Inc., Shanghai, China) were housed individually in Plexiglas cages and checked daily for delivery. Rats were kept on a 12-h light/dark cycle (07∶00–19∶00), constant temperature (22±2°C) and humidity (50–55%) with food and water provided *ad libitum*. The day of delivery was designated as postnatal day (PND) 0. Newborn male pups were randomly assigned to 4 groups: maternal deprivation but non-chronic unpredictable stress group (MD/control, N = 19), non-maternal deprivation but chronic unpredictable stress group (non-MD/CUS, N = 18), MD followed by CUS exposure group (MD/CUS, N = 17) and non-maternal deprivation followed by non-chronic unpredictable stress group (non-MD/control, N = 22). Rats in non-MD/control group received standard housing conditions. Rats in MD/control group were given maternal deprivation from PND1 to PND14. Rats in non-MD/CUS group were given chronic unpredictable stress for 21 days after reaching 10-weeks old. Rats in MD/CUS group were given both maternal deprivation and chronic unpredictable stress. All experiments were conducted in accordance with the National Institutes of Health Guide for the Care and Use of Laboratory Animals and the Chinese legislation on the use and care of laboratory animals. The animal protocol was approved by the Animal Use Committee of Central South University.

### 2. Maternal Deprivation

The MD paradigm was conducted as previously described [20]. Briefly, pups were separated from their mothers for 6 hours daily from PND 1 to PND 14 (the separations occurred at 9∶00–15∶00). To block communication between littermates, each offspring was placed in a single cell (a cage in size of 32 cm×32 cm×14 cm was divided into four cells of the same size) covered with dry sawdust. At the end of the separation period, pups were returned to their maternal cages. All experiments were carried out in a temperature-controlled room (30°C). Rats of the same gender were weaned at PND 21 and housed in groups of four until adulthood (10 weeks).

### 3. Chronic Unpredictable Stress

The CUS paradigm was performed as previously described [21] with minor modifications. Briefly, rats at 10-weeks old were randomly exposed to one of the following stressors once daily for 21 days: water deprivation for 24 hrs, an elevated open platform (10 cm×10 cm, 160 cm in height) for 2 hrs, food deprivation for 24 hrs, restraint stress for 2 hrs, or electric foot shock for 20s (800 mA, 1-s duration, average 1 shock/10s). Stressors were given randomly at different times of the day to establish unpredictability.

### 4. Sucrose Preference Test

Sucrose preference test was used to assess the degree of anhedonia in rats and was performed as previously described [22]. The whole test took 3 days. On the first day, rats were housed individually and given free access to two bottles of sucrose solution (1%, w/v) for 24 hours. On the second day, one bottle of sucrose solution was replaced with water for 24 hours. On the third day, rats were deprived of water and food for 23 h, and during the last 1 h, rats were given free access to two pre-weighed bottles of solution. The position of two bottles was switched. The mass of sucrose and water consumed in both bottles was recorded in grams. The sucrose preference rate was calculated using the following formula: Sucrose preference rate = sucrose consumption (g)/[water consumption (g)+sucrose consumption (g)].

### 5. Forced Swim Test

Forced swim test was used to measure behavioral despair, and was performed following a previously established protocol [23]. Two swimming sessions were conducted: a 15-min pretest on day 1 followed by a 5-min test the next day. During testing, rats were placed individually in a transparent cylinder (21 cm in diameter×46 cm in high) containing water (25°C) with a depth of 30 cm. After swimming for 15 min on day 1, rats were dried with towels and placed back in their home cage. At the end of each trial, the cylinder was emptied and water was refilled to reduce the interference between animals. Twenty-four hrs after the first trial, rats were placed in the swimming apparatus again for a 5-min test trial. A video camera was used to record the experiment. The immobility time (The time a rat spent in keeping its head above water with only slight movements) was calculated.

### 6. Sample Collection

After behavioral tests, rates were anesthetized with intraperitoneal injections of 10% chloral hydrate (30 ml per kg body weight) and were sacrificed by decapitation. Brains were rapidly removed from the skull, cooled in cold physiological saline, and put in a rat brain slicer. According to the rat brain in stereotaxic coordinates, whole NAc tissues were immediately collected. Tissues were snap frozen in liquid nitrogen and kept at −80°C.

### 7. Real-time Reverse Transcription PCR

Quantitative RT-PCR was used to detect DRD1/DRD2 mRNA and miR-504 expression in NAc tissues. Total RNA was extracted using Trizol reagent (Invitrogen, Carlsbad, CA, USA) and reverse transcription was performed using One Step PrimeScript® miRNA cDNA Synthesis Kit (Perfect Real Time) and PrimeScript® RT reagent Kit (Perfect Real Time) (RAKARA, Japan) for miRNA and mRNA, respectively. Real-time quantitative PCR was performed using SYBR® Premix Ex Taq™ II (RAKARA, Japan). The primers are listed in Table 1. miR-504, DRD1 and DRD2 mRNA were normalized to U6 or β-actin.

**Table 1:** Real time-RT-PCR primer sequence.

RNA	primer sequence
miR-504	Forward: 5′-gcggtgaggtagtaggttgtatagtt-3′
	Reverse: provided with the kit
U6	Forward: 5′-gcaaggatgacacgcaaattc-3′
	Reverse: provided with the kit
DRD1	Forward: 5′-cttcgatgtgtttgtgtggttt-3′
	Reverse: 5′-tcttccttcttcaggtcctcag-3′
DRD2	Forward: 5′-cttgatagtcagccttgctgtg-3′
	Reverse: 5′-agggcacgtagaatgagacaat-3′
β-actin	Forward: 5′-cacgatggaggggccggactcatc-3′
	Reverse: 5′-taaagacctctatgccaacacagt-3′

### 8. Western Blot

The rabbit anti-DRD1 and anti-DRD2 antibodies, which are specific for DRD1 and DRD2 protein respectively, were purchased from Santa Cruz Biotechnology (San Diego, CA, USA). The rabbit anti-β-actin antibody, which is specific for β-actin protein, and the horseradish peroxidase-conjugated anti-rabbit IgG were purchased from Sigma-Aldrich (St. Louis, MI, USA). The NAc tissue was homogenized in ice-cold buffer and Western blot was conducted as previously described [24]. Thirty micrograms of total protein was loaded on a 10% SDS-PAGE gel. The same blot was re-probed for β-actin to serve as a loading control. The intensity of each band was quantified with Bio-Rad Quantity One software (Bio-Rad, Hercules, CA, USA).

### 9. Statistical Analysis

The results were presented as M±SD. Data analysis was performed using the statistical software SPSS 17.0. Mean comparisons were performed using a two-way ANOVA with MD (non-MD and MD) and CUS (non-CUS and CUS) as the factors. Pearson correlation analysis was used to evaluate possible associations between miR-504, DRD1/DRD2 gene expression and stress-induced behavior. A *p*<0.05 was considered statistically significant.

## Results

### 1. Maternally Deprived Rats Show Behavioral Vulnerability to CUS

Behavioral data obtained from sucrose preference test and forced swim test were presented in Table 2. In the sucrose preference test, a significant main effect of MD (F = 7.45, *p*<0.05) was observed. The sucrose preference rate was significantly lower in MD rats than in non-MD rats (*p*<0.05). Moreover, a significant interaction between MD and CUS on the sucrose preference rate (F = 4.76, *p*<0.05) was observed. The sucrose preference rate in MD/CUS rats was significantly lower than that in MD/control rats (post hoc, *p*<0.05), non-MD/CUS rats (post hoc, *p*<0.05), or non-MD/control rats (post hoc, *p*<0.05). No significant group differences on sucrose preference rate among MD/control rats, non-MD/CUS rats, and non-MD/control rats (post hoc, *ps*>0.05) were observed.

**Table 2:** The comparison of behavioral indexes in sucrose preference test, forced swim test and the body weight among groups (M±SD).

group	sucrose preference rate	immobility time (s)	body weight (g)
non-MD/Control	0.58±0.20	99.46±27.78	320.50±50.66
MD/Control	0.55±0.20	111.87±58.16	198.33±29.37*
non-MD/CUS	0.63±0.27	122.94±31.83	252.42±28.92
MD/CUS	0.38±0.22*^Δ#^	177.24±57.01*^Δ#^	183.65±11.23*Δ

Notes: * compared with non-MD/Control group, *p*<0.05;

Δ compared with non-MD/CUS group, *p*<0.05;

# compared with MD/Control group, *p*<0.05.

In the forced swim test, significant main effects of MD (F = 10.31, *p*<0.05) and CUS (F = 18.29, *p*<0.05) were observed. The immobility time in MD rats was significantly longer than that in non-MD rats (*p*<0.05). A significant interaction between MD and CUS on the immobility time (F = 4.06, *p*<0.05) was observed. Rats in MD/CUS group had significantly longer immobility time than MD/control rats (post hoc, *p*<0.05), non-MD/CUS rats (post hoc, *p*<0.05), or the non-MD/control rats (post hoc, *p*<0.05), but no significant group differences on immobility time were found among the other three groups (post hoc, *ps*>0.05) (Table 2).

Body weight was measured in all rats. A significant main effect of MD (F = 40.09, *p*<0.05) on body weight was observed. The body weight of MD rats was significantly lighter than that of non-MD rats (*p*<0.05). Moreover, a significant MD×CUS interaction on the body weight (F = 7.53, *p*<0.05) was observed. Rats in the MD/CUS group had significantly lower body weights compared with non-MD/CUS rats (post hoc, *p*<0.05), or non-MD/control rats (post hoc, *p*<0.05). The body weights of MD/control rats was significantly lighter than that of non-MD/control rats (post hoc, *p*<0.05), whereas body weights of non-MD/CUS rats showed no significant differences from rats in the other two groups (Table 2).

### 2. Effect of MD on miR-504 and DRD1 Gene Expression in the NAc in Adult Rats

Quantitative RT-PCR showed that there were no significant differences in U6 and β-actin mRNA levels among MD/control, non-MD/CUS and/or MD/CUS rats. Significant main effect of MD (F = 32.48, *p*<0.05) on miR-504 expression was observed. The miR-504 expression in MD rats was significantly higher than that in non-MD rats (*p*<0.05). A significant MD×CUS interaction on miR-504 expression (F = 4.38, *p*<0.05) was observed. The miR-504 expression in MD/CUS rats was significantly higher than that in MD/control rats (post hoc, *p*<0.05), non-MD/CUS rats (post hoc, *p*<0.05), or non-MD/control rats (post hoc, *p*<0.05). MiR-504 expression in MD/control rats was significantly higher than that in non-MD/control rats (post hoc, *p*<0.05). The expression of miR-504 in non-MD/CUS rats exhibited no significant differences from rats in the other two groups (Table 3).

**Table 3:** The comparison of miR-504, DRD1 mRNA, DRD1 protein expression among groups in NAc (M±SD).

group	miR-504	DRD1 mRNA	DRD1 protein
non-MD/Control	0.013±0.004	0.028±0.010	1.131±0.211
MD/Control	0.019±0.005*	0.015±0.004*Δ	0.995±0.361
non-MD/CUS	0.015±0.002	0.027±0.008	1.255±0.328
MD/CUS	0.029±0.007*^Δ#^	0.015±0.006*Δ	0.558±0.296*^Δ#^

Notes: * compared with non-MD/Control group, *p*<0.05;

Δ compared with non-MD/CUS group, *p*<0.05;

# compared with MD/Control group, *p*<0.05.

A significant main effect of MD on DRD1 mRNA expression in the NAc of rats (F = 23.90, *p*<0.05) was observed. DRD1 mRNA expression in MD rats was significantly lower than that in non-MD rats (*p*<0.05). No significant main effect of CUS and MD×CUS on DRD1 mRNA expression (F = 0.062, *p*>0.05; F = 0.067, *p*>0.05) were observed. The DRD1 mRNA expression in MD/control and MD/CUS rats was significantly lower than that in non-MD/CUS and control rats (post hoc, *p*<0.05). DRD1 mRNA expression in non-MD/CUS rats showed no significant differences from rats in the non-MD/control group (Table 3).

**Figure 1:**
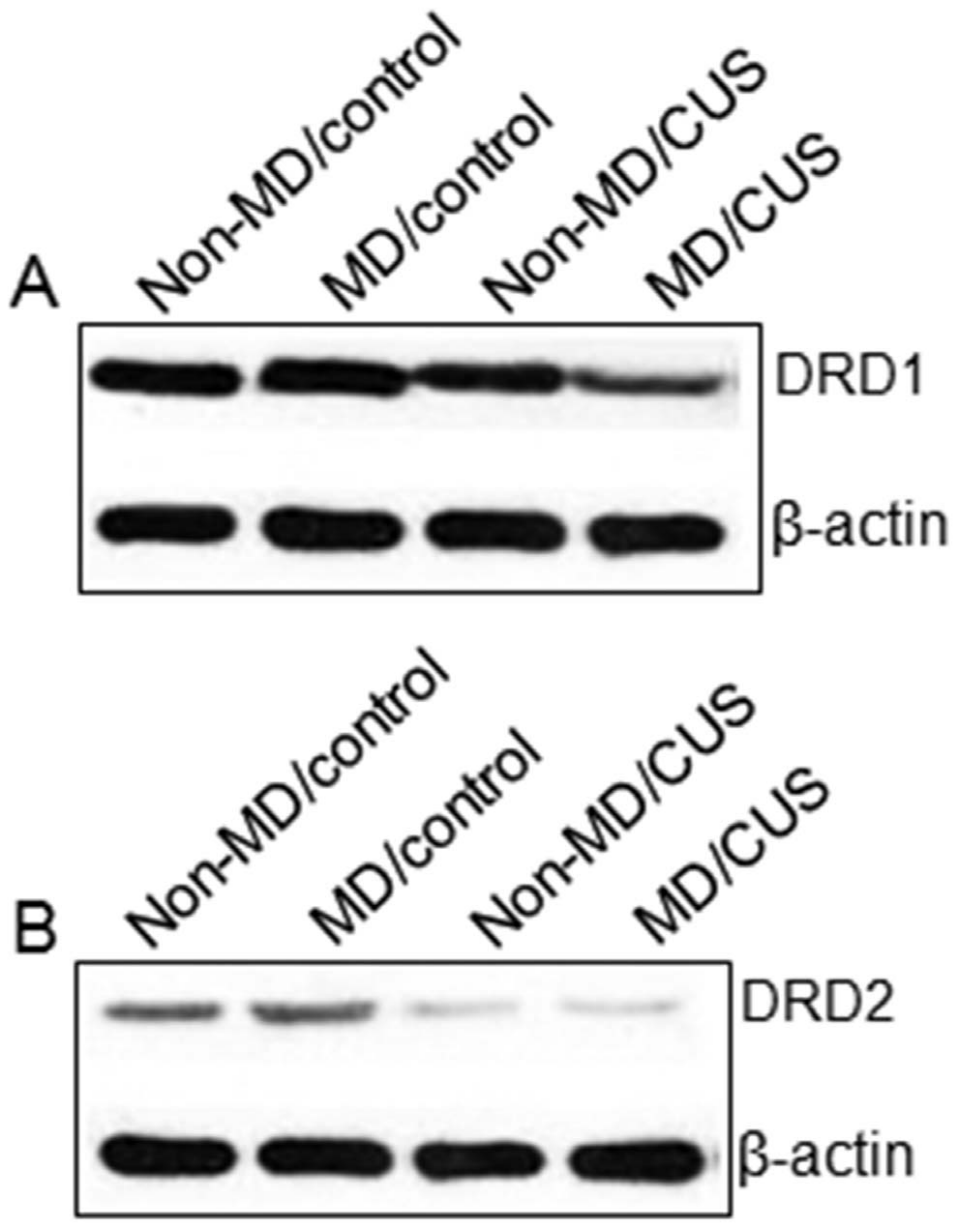
Western blot of DRD1, DRD2 and β-actin among groups. A) Western blot of DRD1 and β-actin among groups. B) Western blot of DRD2 and β-actin among groups.

The DRD1 protein expression in the homogenate of NAc was measured by Western blot (Fig. 1A). A significant main effect of MD (F = 11.23, *p*<0.05) on DRD1 protein expression in the NAc of rats was observed. DRD1 protein expression in MD rats was significantly lower than that in non-MD rats (*p*<0.05). A significant MD×CUS interaction on DRD1 protein expression (F = 5.10, *p*<0.05) was observed. DRD1 protein expression in MD/CUS rats was significantly lower than that in MD/control rats (post hoc, *p*<0.05), non-MD/CUS rats (post hoc, *p*<0.05), or non-MD/control rats (post hoc, *p*<0.05), but no significant group differences on DRD1 protein expression were found among the other three groups(post hoc, *ps*>0.05) (Table 3).

### 3. Effect of MD on DRD2 Gene Expression in the NAc in Adult Rats

As shown in Table 4, a significant main effect of MD on DRD2 mRNA expression in the NAc of rats (F = 78.06, *p*<0.05) was observed. DRD2 mRNA expression in MD rats was significantly lower than that in non-MD rats (*p*<0.05). No significant main effect of CUS and MD×CUS on DRD2 mRNA expression in the NAc of adult rats (F = 0.57, *p*>0.05; F = 0.01, *p*>0.05) were observed. DRD2 mRNA expression in MD/control and MD/CUS rats was significantly lower than that in non-MD/CUS and non-MD/control rats (post hoc, *p*<0.05). No significant differences in DRD2 mRNA expression were observed in non-MD/CUS rats compared to rats in the non-MD/control group (Table 4).

**Table 4:** The comparison of DRD2 mRNA and protein expression in the NAc (M±SD).

group	DRD2 mRNA	DRD2 protein
non-MD/Control	0.023±0.008	0.548±0.269
MD/Control	0.004±0.002*Δ	0.337±0.102*
non-MD/CUS	0.021±0.008	0.446±0.159
MD/CUS	0.002±0.002*Δ	0.261±0.071*

Notes: * compared with non-MD/Control group, *p*<0.05;

Δ compared with non-MD/CUS group, *p*<0.05.

The DRD2 protein expression in the homogenate of NAc was measured by Western blot (Fig. 1B). A significant main effect of MD on DRD2 protein expression in the NAc of rats (F = 8.32, *p*<0.05) was observed. DRD2 protein expression in MD rats was significantly lower than that in non-MD rats (*p*<0.01). No significant main effect of CUS and MD×CUS on DRD2 protein expression in the NAc of rats (F = 0.21, *p*>0.05; F = 0.04, *p*>0.05) were observed. The DRD2 protein expression in MD/control and MD/CUS rats was significantly lower than that in non-MD/CUS and non-MD/control rats (post hoc, *p*<0.05). No significant differences in DRD2 protein expression were observed in non-MD/CUS rats compared to rats in the non-MD/control group (Table 4).

### 4. Correlation Analysis between miR-504 and DRD1 mRNA Expression, as well as DRD1 Protein Expression in the NAc and Behavioral Indices in Rats

**Figure 2:**
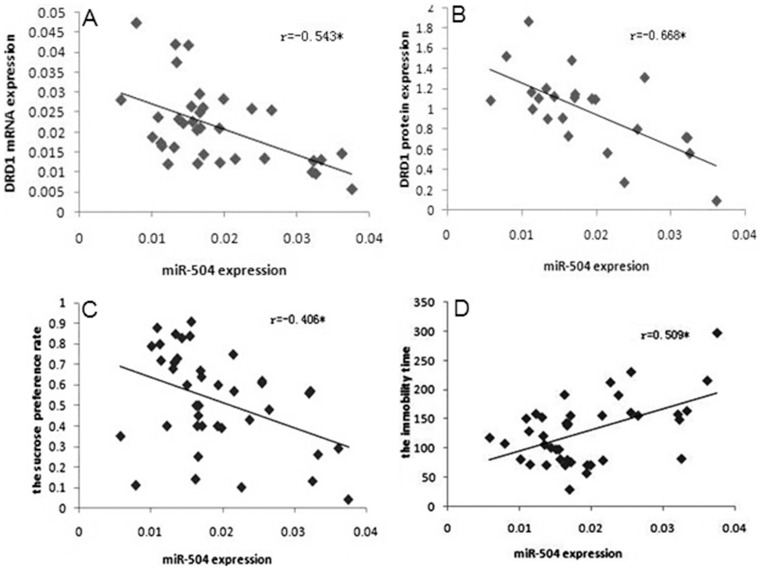
Correlation between miR-504 expression, DRD1 gene expression and depression-like behaviors. A) Correlation between miR-504 expression and DRD1 mRNA expression. B) Correlation between DRD1 mRNA expression and DRD1 protein expression. C) Correlation between miR-504 expression and the sucrose preference rate. D) Correlation between miR-504 expression and the immobility time.

**Figure 3:**
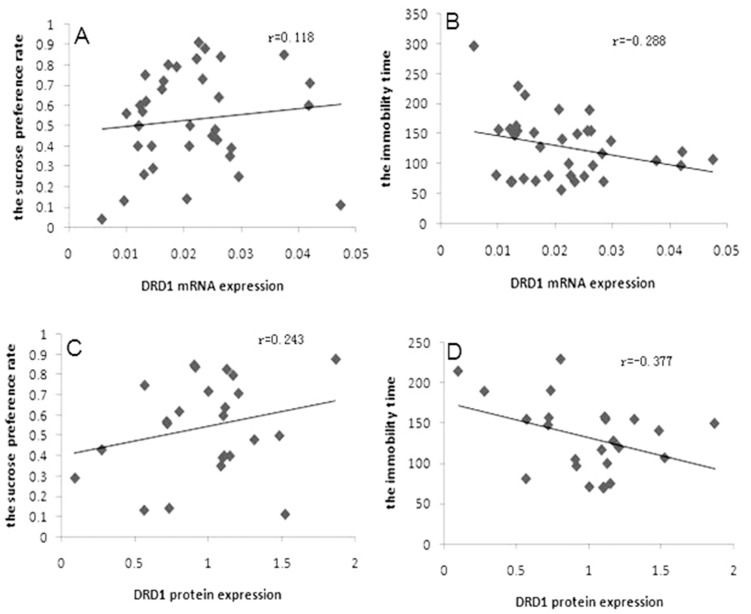
Correlation between DRD1 gene expression and depression-like behaviors. A) Correlation between DRD1 mRNA expression and the sucrose preference rate. B) Correlation between DRD1 mRNA expression and the immobility time. C) Correlation between DRD1 protein expression and the sucrose preference rate. D) Correlation between DRD1 protein expression and the immobility time.

Pearson correlation analysis revealed that miR-504 expression negatively correlated with the level of DRD1 mRNA (Fig. 2A, *p*<0.05), DRD1 protein (Fig. 2B, *p*<0.05) and sucrose preference rate in the sucrose preference test (Fig. 2C, *p*<0.05), while miR-504 expression positively correlated with immobility time in forced swim test (Fig. 2D, *p*<0.05). Unexpectedly, DRD1 mRNA and DRD1 protein expression didn’t significantly correlate with the sucrose preference rate (Fig. 3A, *p*>0.05; Fig. 3C, *p*>0.05, respectively) or the immobility time (Fig. 3B, *p*>0.05; Fig. 3D, *p*>0.05, respectively).

### 5. Correlation Analysis of DRD2 mRNA and Protein Expression in the NAc with Behavioral Indices in Rats

**Figure 4:**
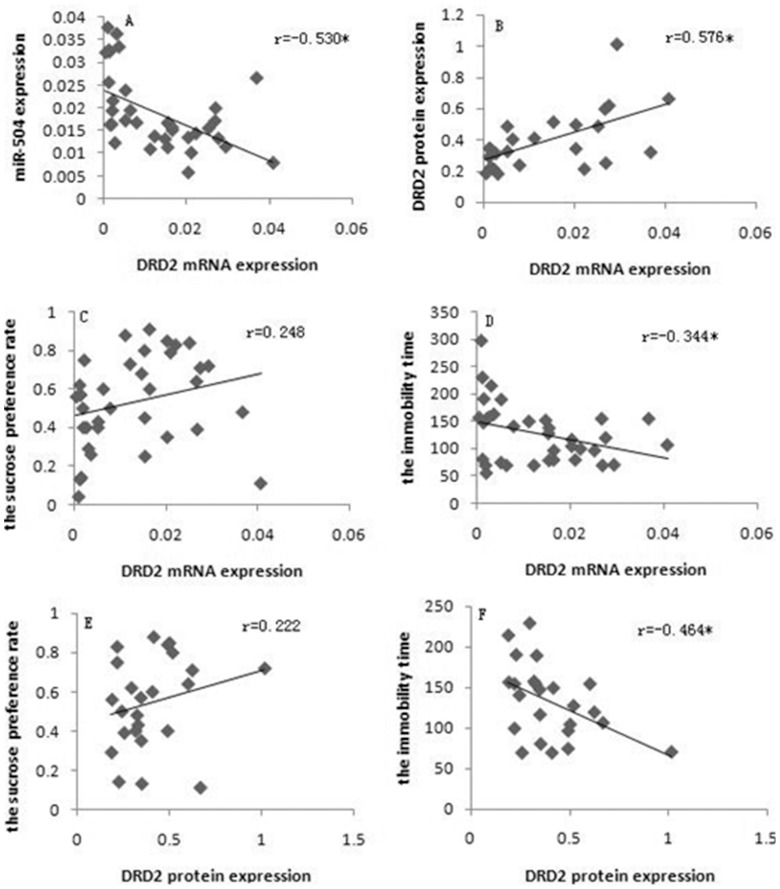
Correlation between DRD2 gene expression and depression-like behaviors. A) Correlation between DRD2 mRNA expression and miR-504 expression. B) Correlation between DRD2 mRNA expression and DRD2 protein expression. C) Correlation between DRD2 mRNA expression and the sucrose preference rate. D) Correlation between DRD2 mRNA expression and the immobility time. E) Correlation between DRD2 protein expression and the sucrose preference rate. F) Correlation between DRD2 protein expression and the immobility time.

Pearson correlation analysis revealed that DRD2 mRNA expression significantly correlated with miR-504 expression (Fig. 4A, *p*<0.05) and DRD2 protein (Fig. 4B, *p*<0.05). DRD2 mRNA and DRD2 protein expression didn’t significantly correlate with the sucrose preference rate (Fig. 4C, *p*>0.05; Fig. 4E, *p*>0.05, respectively), while both did negatively significantly correlate with the immobility time (Fig. 4D, *p*<0.05; Fig. 4F, *p*<0.05, respectively).

## Discussion

In humans, adverse events in early life are major environmental factors associated with increased sensitivity to developing mood disorders [2]. Early life stresses are able to affect the immature neural circuitry and increase susceptibility to depression, whereas stressful events that occur in adulthood may only activate or enhance the emergence of such symptoms. In the present study, the decrease in sucrose preference rate and increase in immobility time during sucrose preference test and forced swim test reflect an increase in anhedonia and despair like-behaviors. MD/control or non-MD/CUS rats did not have changes in sucrose preference rate or the immobility time, but MD following by CUS significantly decreased the sucrose preference rate and increased the immobility time in stressed rats compared with MD/control and non-MD/CUS. Thus, our data suggested that early stress enhances susceptibility to depression during adulthood.

It is well known that depression is associated, in part, with dysregulation of the mesolimbic dopaminegeric (DA) system. The mesolimbic DA system, especially, the DA receptors are thought to play an important role in the development of depression [25], [26]. Furthermore, previous research suggested that early life events may prime the mesolimbic DA system for dysregulation in adult life [27]. For example, MD in early life was found to increased DA levels in the NAc of rats/mice during adulthood [28], as well as increasing sensitivity to depression [29]. However, our study demonstrated that MD alone decreased DRD1 and DRD2 mRNA and protein levels in the NAc in adult rats, suggesting that MD has a long-term effect on the DRD1 and DRD2 gene expression in the NAc. However, no significant depressive behaviors were observed in MD/control rats. Moreover, there is no significant correlation with between DRD1 mRNA, DRD1 protein expression, the sucrose preference rate, and the immobility time. Inconsistent with previous studies, our finding suggested a limited role of DRD1 in the depressive behaviors of stressed rats. We hypothesized that the inconsistency may be caused by differences in stress models. In contrast, DRD2 mRNA and DRD2 protein expression negatively correlated with immobility time, suggesting that DRD2 may play a more important role than DRD1 in the development of stress-induced depression.

A novel finding in this study is that miR-504 expression is significantly associated with depressive behaviors in stressed rats. We first demonstrated that MD upregulated miR-504 expression in the NAc and that MD followed by CUS more significantly increased miR-504 expression compared to MD/control or non-MD/CUS. In contrast, no change in miR-504 expression was observed in the NAc of rats treated with CUS alone. In fact, both the body weight and behavioral tests showed that the CUS alone paradigms did not induce depression in this study. These findings indicated that if a rat was exposed to CUS alone but did not develop a depression-like phenotype, miR-504, DRD1, and DRD2 expressions were not altered in the NAc. Therefore, the observed alterations in miR-504, DRD1, and DRD2 expressions should only be associated with depression-like phenotype. Furthermore, the upregulation of miR-504 negatively correlated with the sucrose preference rate and positively correlated with the immobility time. Therefore, elevated miR-504 expression may be responsible for the development of stress susceptibility of rodents exposed to MD during early postnatal period. A previous in vitro study showed that miR-504 could up-regulate DRD1 expression by binding directly to the 3′-UTR of the human DRD1 gene [19]. In this study, miR-504 expression negatively correlated with the expression of DRD1 mRNA, which provided indirect evidence for the regulation of miR-504 on DRD1 expression. However, DRD1 mRNA expression did not significantly correlate with DRD1 protein expression, and DRD1 protein levels showed no association with depressive behaviors. Our findings suggested that the involvement of miR-504 in stress-induced depressive behaviors is not mediated by the regulation of DRD1 expression in the NAc. In contrast, other mechanisms involved in the regulation of DRD1 translation or miR-504 could have regulated the expression of other genes responsible for the role of miR-504 in stress-induced depression. Our study also suggests that the mRNA level and the translation rate of the DRD1 gene may be regulated by stressors in this study through undefined mechanisms. Furthermore, because miR-504 is associated with the X chromosome and only male rats were used in this study, the modulation of DRD1 expression by miR-504 may play a role in the influence of sex on stress. Further studies are required to determine whether stressors affect post-translational modification of DRD1 protein and whether that leads to subsequent DRD1 protein stability.

It has been demonstrated that stress can increase the expression of cAMP response element binding (CREB) protein in the NAc [30], but its activity is regulated by DA through both DRD1 and DRD2 receptors [31]. In this study, we demonstrated that miR-504 expression negatively correlated with DRD2 mRNA level, while DRD2 mRNA level positively correlated with its protein level in the NAc. Currently, no direct target of miR-504 on 3′-UTR of DRD2 gene has been identified. Whether miR-504 targets some genes which directly or indirectly regulate DRD2 mRNA transcription require further studies. A previous study demonstrated that miR-504 directly represses p53 protein expression, thereby negatively regulating p53-mediated apoptosis, which has been found in depressed humans and/or animals [32]. Increased miR-504 may possibly repress p53 expression and induce apoptosis in dopaminergic neurons, which results in a decrease in the number of dopaminergic neurons and subsequent decrease in DRD1/DRD2 expression.

In conclusion, our data suggest that MD enhances behavioral vulnerability to stressors during adulthood through the upregulation of miR-504 expression in the NAc. miR-504 may mediate the downregulation of DRD1 and DRD2 expression in the nucleus accumbens, but DRD2 not DRD1 is involved in stress-induced depression in the animal models tested in this study.
